# Prediction of radiation pneumonitis after definitive radiotherapy for locally advanced non-small cell lung cancer using multi-region radiomics analysis

**DOI:** 10.1038/s41598-021-95643-x

**Published:** 2021-08-10

**Authors:** Daisuke Kawahara, Nobuki Imano, Riku Nishioka, Kouta Ogawa, Tomoki Kimura, Taku Nakashima, Hiroshi Iwamoto, Kazunori Fujitaka, Noboru Hattori, Yasushi Nagata

**Affiliations:** 1 Department of Radiation Oncology, Graduate School of Biomedical Health Sciences, Hiroshima University, 1-3-2 Kagamiyama, Higashihiroshima, Hiroshima 734-8551 Japan; 2 Medical and Dental Sciences Course, Graduate School of Biomedical & Health Sciences, Hiroshima University, Hiroshima, Japan; 3 School of Medicine, Hiroshima University, Hiroshima, Japan; 4 Division of Radiation Oncology Kochi Medical School, Department of Radiology, Kochi University, Kochi, Japan; 5 Department Molecular and Internal Medicine, Graduate School of Biomedical Health Sciences, Hiroshima University, Hiroshima, Japan; 6 Hiroshima High-Precision Radiotherapy Cancer Center, Hiroshima, Japan

**Keywords:** Medical research, Oncology

## Abstract

To predict grade ≥ 2 radiation pneumonitis (RP) in patients with locally advanced non-small cell lung cancer (NSCLC) using multi-region radiomics analysis. Data from 77 patients with NSCLC who underwent definitive radiotherapy between 2008 and 2018 were analyzed. Radiomic feature extraction from the whole lung (whole-lung radiomics analysis) and imaging- and dosimetric-based segmentation (multi-region radiomics analysis) were performed. Patients with RP grade ≥ 2 or < 2 were classified. Predictors were selected with least absolute shrinkage and selection operator logistic regression and the model was built with neural network classifiers. A total of 49,383 radiomics features per patient image were extracted from the radiotherapy planning computed tomography. We identified 4 features and 13 radiomics features in the whole-lung and multi-region radiomics analysis for classification, respectively. The accuracy and area under the curve (AUC) without the synthetic minority over-sampling technique (SMOTE) were 60.8%, and 0.62 for whole-lung and 80.1%, and 0.84 for multi-region radiomics analysis. These were improved 1.7% for whole-lung and 2.1% for multi-region radiomics analysis with the SMOTE. The developed multi-region radiomics analysis can help predict grade ≥ 2 RP. The radiomics features in the median- and high-dose regions, and the local intensity roughness and variation were important factors in predicting grade ≥ 2 RP.

## Introduction

Chemoradiotherapy (CRT) is the standard treatment for unresectable locally advanced non-small cell lung cancer (NSCLC). However, CRT for NSCLC carries the risk of radiation pneumonitis (RP)^1^. Although the development of immunotherapy has been shown to significantly improve survival after CRT^2^, immunotherapy cannot be continued when RP develops. Previous studies have developed many indicators for predicting grade 3 RP as a serious adverse event^3–7^. However, it is extremely important to predict grade ≥ 2 RP because immunotherapy cannot be continued if it occurs.

Various indicators have been shown to predict grade 2 and 3 RP by CRT for NSCLC, including sex, smoking status, tumor location, age, and pulmonary comorbidity^8,9^. These patient backgrounds should be carefully considered when predicting RP. Another approach for predicting RP is dosimetric predictors. The mean lung dose or the volume of the lung receiving > 20 Gy (V20) has been reported as the best correlated predictor of RP^8,9^. We previously reported the importance of reducing the high-dose area by analyzing NSCLC patients who received three-dimensional conformal radiotherapy (3D-CRT) or intensity-modulated radiotherapy (IMRT)^10^. These dose-volume histogram (DVH) parameters have been considered individually; however, these factors need to be considered comprehensively. In addition, there are individual differences in the risk of developing RP among patients with the same dosimetric predictors, probably because of differences in patient backgrounds. Therefore, predictive indicators that consider both individual patient background factors and all types of dosimetric factors are needed.

Recently, radiomics has been proposed to explore the correlation between medical images, underlying genetic information, and other characteristics^11^. It has been used to classify patients and evaluate their risk in order to tailor and customize prescribed oncological treatments. Texture features from computed tomography (CT) images are useful for differentiating lung phenotypes resulting from patients’ lung backgrounds^12^. Krafft et al. proposed a predictive model for RP toxicity after radiotherapy using pretreatment CT-based radiomics features extracted from the whole-lung volume^13^. They proposed a predictive model with CT-based radiomics features extracted from only the whole-lung region, which reflects patients’ lung background but not dosimetric factors. We hypothesized that radiomics analysis can be improved by increasing the region of interest (ROI), which reflects all kinds of dosimetric factors. Previous studies have reported the usability of increasing the radiomics features by adding the ROI in the outer region of the tumor to improve prediction accuracy^14,15^. These approaches showed that the imaging features from multi-region radiomics analysis were useful for predicting treatment outcomes; however, these ROIs did not reflect dosimetric factors, and no report has utilized this method to predict RP.

In the current study, we proposed a method of multi-region radiomics analysis that can reflect both patients’ lung background and all kinds of imaging and dosimetric factors in the imaging and dosimetric-based segmentations to predict grade ≥ 2 RP in locally advanced NSCLC.

## Materials and methods

### Patients

The eligibility criteria for this study were as follows: histologically confirmed NSCLC; clinical stage II, IIIA, IIIB, or IVA according to the 8th TNM staging system of the International Union Against Cancer (UICC); no distant organ metastasis diagnosed by CT, magnetic resonance imaging (MRI), or positron emission tomography CT (PET-CT); had been irradiated using three-dimensional conformal radiotherapy (3D-CRT); had been followed until the onset of RP of grade 2 or higher, or for more than 6 months after the completion of treatment; had no previous history of radiotherapy to the chest; RP grade was evaluated by a radiation oncologist; and digital imaging and communications in medicine (DICOM) data were available. The Hiroshima University Review Board approved this retrospective study (E-1656). The need for informed consent was waived owing to the retrospective nature of the study by Hiroshima University Review Board. The methods were performed according to relevant guidelines and regulations.

### Image acquisition

**Figure 1:**
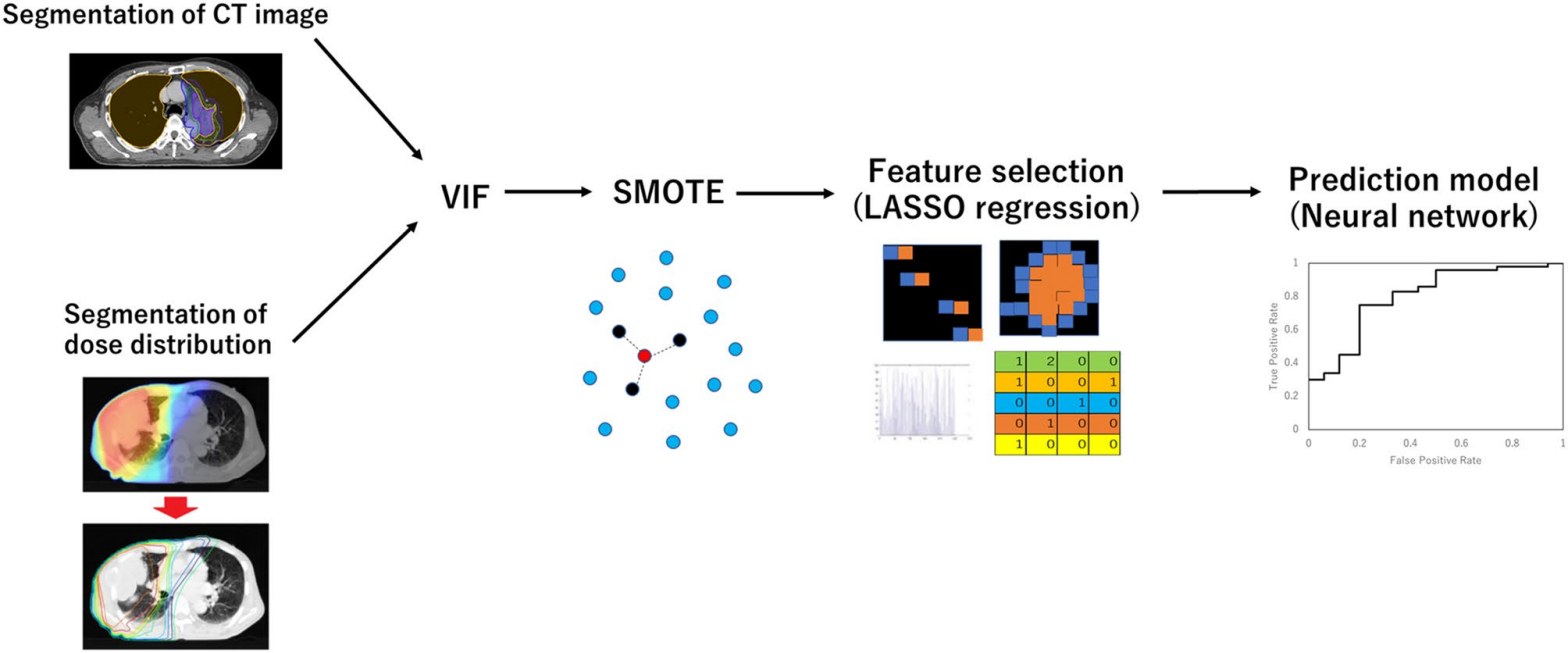
Process of the radiomics analysis and creation of the prediction model.

Figure 1 shows the workflow of this study. CT imaging was performed during free breathing using a CT scanner (Lightspeed RT16, GE Healthcare; Little Chalfont, UK). The slice thickness and slice interval were 2.5 mm.

### Radiotherapy treatment

Radiotherapy was performed with 3D radiotherapy treatment planning for all patients. The gross tumor volume (GTV) included primary tumor and lymph node (LN) metastasis. The clinical target volume (CTV) for the primary lesion and LNs was defined as the GTV with a 3–5 mm and 0–3 mm margin in all directions, respectively. Margins of 5–10 mm were added to the CTV to determine the planning target volume (PTV). Elective nodal irradiation was omitted in all patients. Target delineation was confirmed by the same radiation oncologist according to the same treatment protocol for all patients. A total irradiation dose of 60–74 Gy was administered to the PTV. The dose calculation algorithm used was a collapsed cone convolution superposition/convolution algorithm (CCCS), which is available in Pinnacle3 (Philips Radiation Oncology Systems, Fitchburg, WI).

### Follow-up

Patients were followed up every month after treatment completion until 6 months, and every 3 months thereafter. Patients received a chest X-ray every month and chest-to-pelvis CT at 1-, 3-, and 6-month follow-up visits and every 3–6 months thereafter. RP was evaluated using the Common Terminology Criteria for Adverse Events (CTCAE) version 5.0^16^. More specifically, we defined grade 2 RP as the use of any drug for RP symptoms within 12 months of the end of radiotherapy.

### Radiomics analysis

First, normalization (z-score transformation) of image intensity was performed on the whole image to transform arbitrary CT values into standardized intensity ranges, thereby avoiding heterogeneity bias. Then, the entire CT imaging dataset was analyzed to extract textural features from the segmentations of the radiotherapy plans and dose distribution.

The current study proposed two radiomics analyses: one was the whole-lung radiomics analysis proposed by Krafft et al. in which radiomics features are extracted only in the whole-lung region^13^, and the other was multi-region radiomics analysis in which radiomics features are extracted in the imaging- and dosimetric-based segmentation. CT image segmentation was defined as imaging-based segmentation, as shown in Table 1. In addition to the GTV, CTV, and PTV radiomics features that were used in treatment planning, the shell radiomics features of the region around the GTV, CTV, and PTV, and inner radiomics features of the region that excluded the tumor boundaries, were extracted.

**Table 1:** Imaging-based segmentation list in multi-region radiomics analysis.

Imaging-based segmentation list			
GTV	GTV-2 mm	GTV + 5 mm	GTV + 10 mm
	(GTV + 5 mm)-GTV	(GTV + 10 mm)-GTV	
CTV	CTV-GTV		
PTV	PTV + 5 mm	PTV + 10 mm	PTV + 20 mm
	PTV-GTV		
	(PTV + 5 mm)-PTV	(PTV + 10 mm)-PTV	(PTV + 20 mm)-(PTV + 5 mm)
Lung	Lung-GTV	Lung-PTV	
	Lung-(GTV + 5 mm)		
	Lung-(PTV + 5 mm)	Lung-(PTV + 10 mm)	Lung-(PTV + 20 mm)

*“ROI-Xmm”* indicates the inner tumor region of ROI minus X mm of the outer edge. *ROI* is the region of interest, which represents the GTV, CTV, PTV, or whole lung in the current study. *“ROI + Xmm”* indicates the expanded region from the ROI. *“(ROI + Xmm)-ROI”* indicates the shell region within X mm around the ROI. *GTV* gross tumor volume, *CTV*, clinical target volume, *PTV* planning target volume.

RP occurs outside the tumor, and lungs that receive low doses (5 or 20 Gy) are associated with RP^17,18^. Thus, the radiomics features of the lungs overlapping with the outside of the GTV and PTV were analyzed. Moreover, the target received a higher dose, and normal lungs or whole lungs that received lower to higher doses were added to the analysis as dosimetric-based segmentation, as shown in Table 2.

**Table 2:** Dosimetric-based segmentation list in multi-region radiomics analysis.

Dosimetric-based segmentation list
Lung received 5–60 Gy or higher : Lung ∩ 5–60 Gy
(Lung-GTV) received 5–60 Gy or higher: Lung-GTV ∩ 5–60 Gy
Lung-PTV received 5–60 Gy or higher: Lung-PTV ∩ 5–60 Gy
PTV received 40–60 Gy or higher: PTV ∩ 40–60 Gy
(PTV + 5 mm) received 30–60 Gy or higher: PTV + 5 mm ∩ 30–60 Gy
(PTV + 10 mm) received 20–60 Gy or higher: PTV + 10 mm ∩ 20–60 Gy
(PTV + 20 mm) received 10–60 Gy or higher: PTV + 20 mm ∩ 10–60 Gy

*“ROI ∩ XX Gy”* indicated the ROI received *XX* Gy or higher.

*GTV* gross tumor volume, *CTV* clinical target volume, *PTV* planning target volume.

Feature extraction was performed using Pyradiomics, an open-source package in Python^19^. A detailed list of radiomics features is provided in Table S1 and Table S2.

A list of 50 quantitative features, including 21 first-order features, 13 shape features, and 93 texture analysis features (such as gray-level size zone matrix [GLSZM] features and gray-level run length matrix [GLRLM] features) were extracted. Moreover, the CT images leave the image unchanged as the original image, and these are preprocessed with a wavelet imaging filter. The wavelet filter has low-pass (L) and high-pass (H) filters. The decompositions are constructed in the x-, y-, and z-directions. For example, HLL is then interpreted as the high-pass sub-band, resulting from the directional filtering of X with a high-pass filter in the x-direction, a low-pass filter in the y-direction, and a low-pass filter in the z-direction. In the current study, eight wavelet decompositions (HLL, LHL, LHH, LLH, HLH, HHH, HHL, and LLL) were performed. Each feature was computed separately for each preprocessing step. A total of 837 features were analyzed for each segmentation in this study.

### Feature selection

Interactions between radiomics features were evaluated using variance inflation factor (VIF) analysis. We used the VIF to remove factors of VIF > 10. Next, the least absolute shrinkage and selection operator (LASSO) regression model, which is suitable for the regression of high-dimensional data for whole-lung radiomics analysis and multi-region radiomics analysis, was used with MATLAB code (MathWorks; Natick, MA)^20,21^. LASSO performs feature selection during model construction by penalizing the respective regression coefficients. As this penalty is increased, more regression coefficients shrink to zero, resulting in a more regularized model. The most significant predictive features were selected from among all the candidate features in the training set with tenfold cross-validation.

### Subsampling

There were 45 and 32 patients with grade ≥ 2 and ≤ 1 RP, respectively. To prevent overfitting by the unbalanced ratio, the current study used the synthetic minority over-sampling technique (SMOTE), which is an enhanced sampling method. It interpolates data based on the Euclidean distance for variables. Thus, SMOTE increases the representation of the minority group in the resulting dataset while reflecting the structure of the original dataset^22^. The robustness of a variety of classifiers using SMOTE analysis has been introduced in previous studies^23^. In the current study, SMOTE was used for constructing the prediction model on the selected training dataset before LASSO analysis.

### Prediction model

The objective of this study was to stratify patients into two classes using different machine learning (ML) classifiers. In this regard, patients with RP grade ≥ 2 were labeled as 1, and patients with RP grade < 2 were labeled as 0. This was repeated to classify patients according to their stage. The ML-based classification was performed using a neural network. As shown in Fig. S1, all patients were randomly partitioned into a training/validation set (70% of patients), or testing set (30% of patients). The training/validation set was increased to 78 from 54 patients by the SMOTE. The ratio of RP labels was the same for the training and test datasets. We tested different combinations of feature selection and classification methods to find the best predictive models for these classifications. Classifiers were trained using the fivefold cross-validation method, and their predictive performance was evaluated using the area under the receiver operator characteristic (ROC) curve (AUC). As shown in Fig. S1, the training-validation-testing processes were repeated five times for the fivefold cross-validation. The predictive performance of all models was compared based on the mean AUC.

## Results

**Figure 2:**
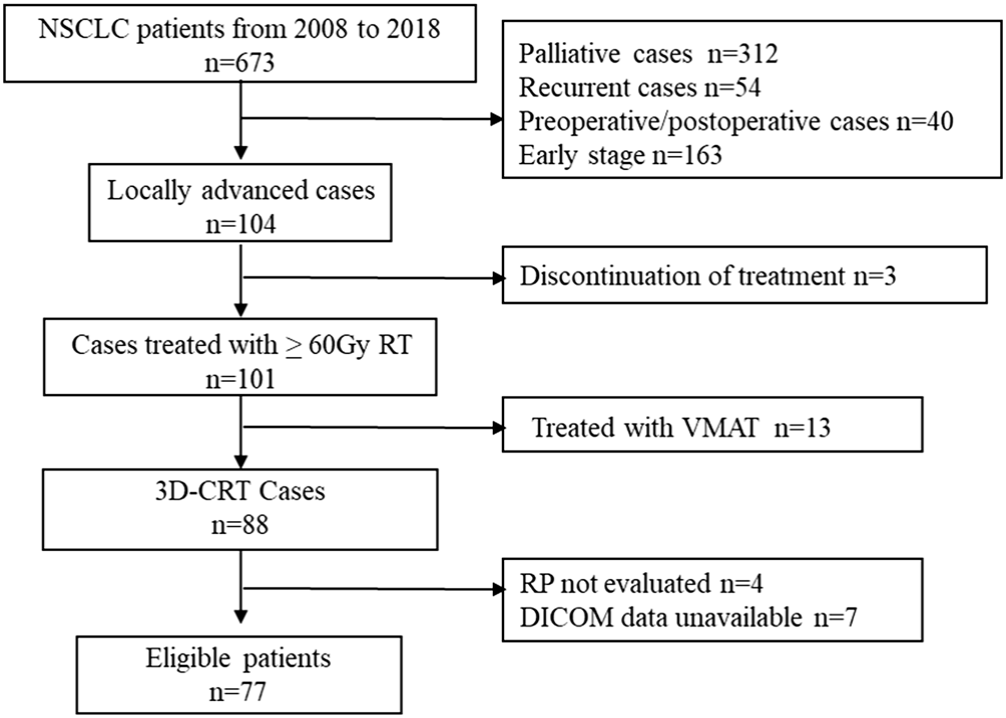
Enrollment characteristics of the study participants. *NSCLC* non-small cell lung cancer, *RT* radiotherapy, *3D-CRT* three-dimensional conformal radiotherapy, *VMAT* volumetric-modulated arc therapy, *RP* radiation pneumonitis, *DICOM* Digital imaging and communications in medicine.

A flowchart of the study population is shown in Fig. 2. A total of 77 patients were included in the radiomics analysis in this study. The characteristics of the patients and their tumors are shown in Table S3. Table S4 shows the characteristics of the patients for training/validation and test dataset. In total, 32 of 77 patients (42%) developed grade ≥ 2 RP.

From the radiomics analysis, a total of 49,383 features were extracted from the CT images. By VIF-based feature reduction, the number of radiomic features was reduced from 49,383 to 32,541. In the whole-lung radiomics analysis, four features were selected using the LASSO regression model, as shown in Table 3 and Fig. S2. These features included the minimum and median CT numbers and the magnitude of the fineness and coarseness of the texture. All radiomics features used a wavelet filter. Features with non-uniformity, local intensity roughness, and a higher ratio of regions with high pixel numbers were selected.

**Table 3:** Selected features by least absolute shrinkage and selection operator regression in the whole-lung-region radiomics analysis.

ROI	Imaging filter	Feature	
Lung	Wavelet-LLH	firstorder	Minimum
Lung	Wavelet-HHL	firstorder	Median
Lung	Wavelet-HHH	glcm	Correlation
Lung	Wavelet-HHL	glcm	Correlation

In the multi-region radiomics method, 13 features were selected using the LASSO regression model, as shown in Table 4 and Fig. S3. Out of 13 features, 5 features were selected from the shape analysis of the dosimetric segmentation, 3 features were selected from the statistical analysis, and 5 features were selected from the texture analysis. From the shape analysis, five shape features from the median-dose (20–30 Gy) and high-dose (60 Gy) regions from the overlapping region of dosimetric segmentation and lung were selected. For the statistical and texture analysis, four features were selected from the whole lung and normal-lung regions, two features were selected from the dosimetric segmentation, and two features were selected from the overlapping region of dosimetric segmentation and lung.

**Table 4:** Selected features by least absolute shrinkage and selection operator regression in the multi-region radiomics analysis.

ROI	Imaging filter	Feature	
Lung-GTV	Wavelet-LLH	glcm	JointAverage
Lung-GTV	Wavelet-LLH	firstorder	Minimum
Lung-GTV	Wavelet-HHL	gldm	Small Dependence Low Gray Level Emphasis
Lung-PTVad20	Wavelet-LLH	ngtdm	Strength
5.00 Gy	Wavelet-LHL	firstorder	Skewness
20.00 Gy	Wavelet-LHL	firstorder	Skewness
30 Gy ∩ Lung-GTV	Wavelet-HLL	gldm	Gray Level Non-Uniformity
50 Gy ∩ Lung-GTV	wavelet-LHL	glszm	Large Area High Gray Level Emphasis
60 Gy ∩ Lung-GTV	Original	Shape	MajorAxis
60 Gy ∩ Lung-GTV	Original	Shape	Flatness
20 Gy ∩ Lung	Original	Shape	MinorAxis
30 Gy ∩ Lung	Original	Shape	MajorAxis
60 Gy ∩ Lung-PTV	Original	Shape	Maximum 3D Diameter

**Figure 3:**
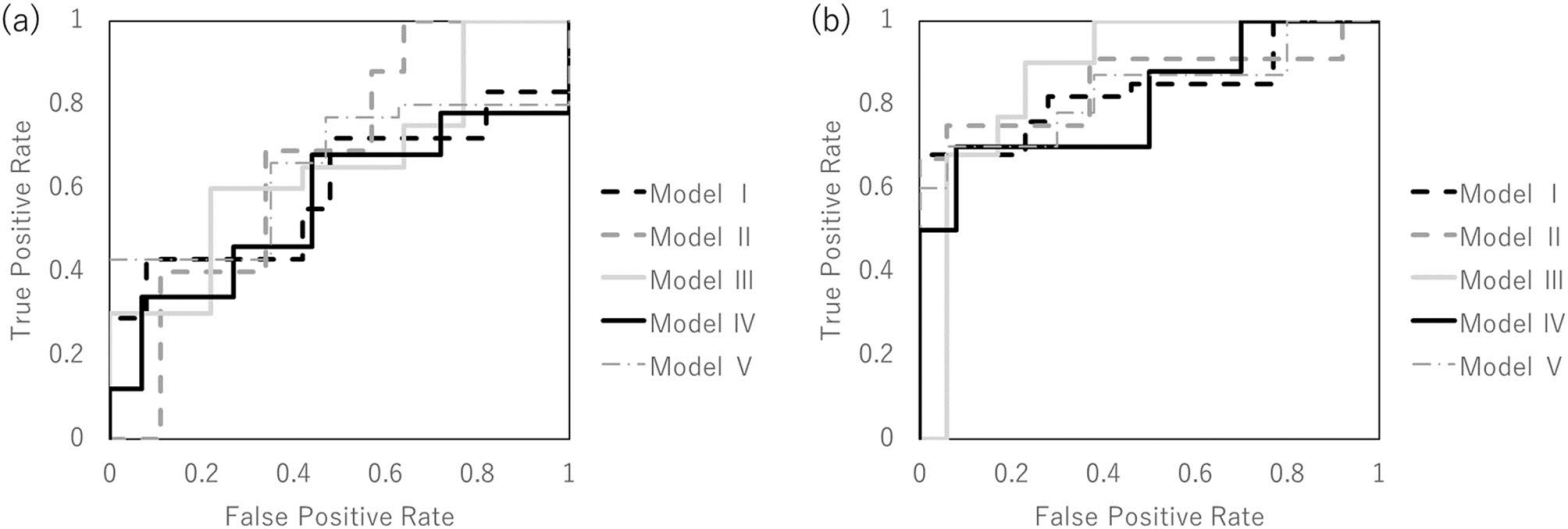
The performance of the predictive model without the synthetic minority over-sampling technique was validated according to the receiver operating characteristic metrics with fivefold cross validation in the whole-lung-region radiomics (**a**) and multi-region radiomics analysis (**b**).

Figure 3a shows the validation of the performance of the predictive model without SMOTE according to ROC metrics with fivefold cross validation in the whole-lung radiomics analysis. Table 5 shows the results of the accuracy, sensitivity, specificity, and AUC of the prediction model in the whole-lung radiomics analysis for the training and testing data. The average accuracy of the five models was 66.8% for the training data. The average accuracy, sensitivity, and specificity of the test data were 66.6%, 50.8%, and 59.0%, respectively. The AUC was 0.60 for the first model, 0.67 for the second model, 0.668 for the third model, 0.56 for the fourth model, and 0.65 for the fifth model. The average AUC with fivefold cross validation was 0.62 ± 0.04.

**Table 5:** Assessment of the predictive performance of the predictive model for training and testing data in the whole-lung radiomics analysis without the synthetic minority over-sampling technique.

	Training (%)	Test (%)
Sensitivity	78.4 (76.9–80.0)	66.6 (58.3–77.8)
Specificity	54.6 (47.1–65.0)	50.8 (40.0–57.1)
Accuracy	66.8 (65.2–71.7)	59.0 (57.9–64.9)
AUC	–	0.62 (0.56–0.67)

AUC, area under the curve.

Next, we applied multi-region radiomics analysis to improve the accuracy of RP prediction. Figure 3b shows that the performance of the predictive model without SMOTE was validated according to the ROC metrics with fivefold cross validation in the multi-region radiomics analysis. Table 6 shows the results of the accuracy, sensitivity, specificity, and AUC of the prediction model in the whole-lung radiomics analysis and the multi-region radiomics analysis for the training and testing data. The average accuracy of the five models was 78.0% for the training data. The average accuracy, sensitivity, and specificity of the test data were 82.7%, 78.4%, and 77.4%, respectively. The average accuracy, sensitivity, specificity, and AUC in the multi-region radiomics analysis were improved compared to those in the whole-lung radiomics analysis.

**Table 6:** Assessment of the predictive performance of the predictive model for training and testing data in multi-region radiomics analysis without the synthetic minority over-sampling technique.

	Training (%)	Test (%)
Sensitivity	82.6 (77.8–91.3)	82.7 (66.7–89.2)
Specificity	71.6 (63.2–88.2)	78.4 (66.7–90.0)
Accuracy	78.0 (73.6–83.3)	77.4 (73.9–82.6)
AUC	–	0.84 (0.81–0.88)

*AUC* area under the curve.

**Figure 4:**
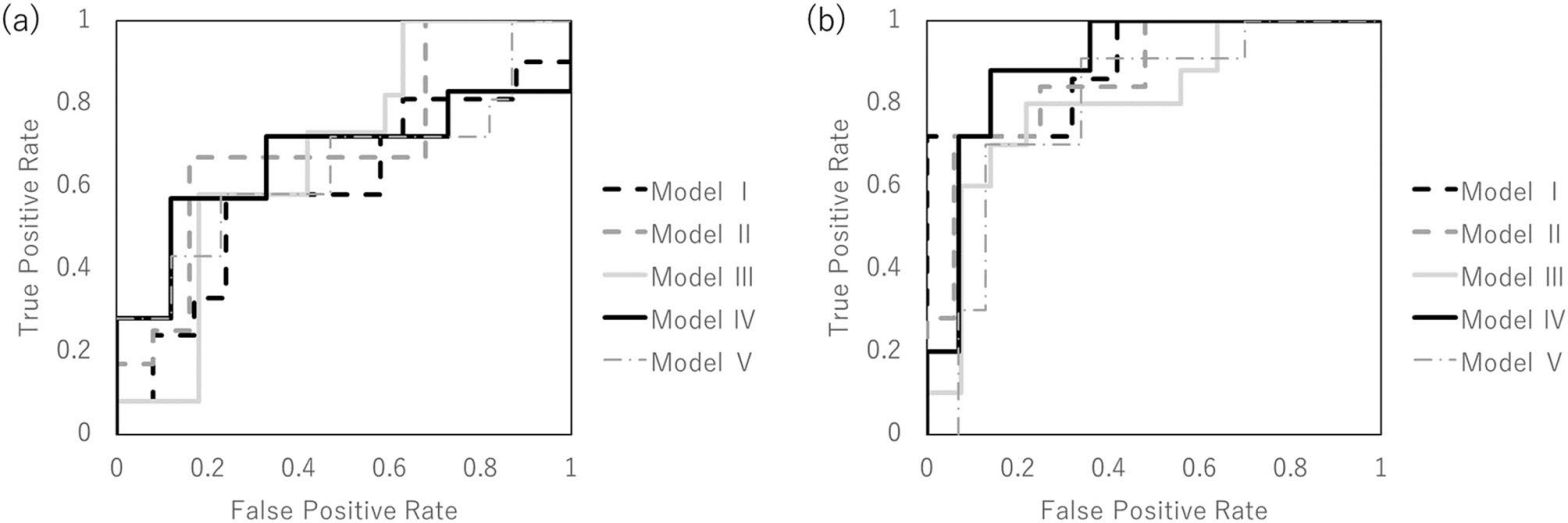
The performance of the predictive model with the synthetic minority over-sampling technique was validated according to the receiver operating characteristic metrics with fivefold cross validation in the whole-lung-region radiomics (**a**) and multi-region radiomics analysis (**b**).

Figure 4 shows the performance of the predictive model with SMOTE according to ROC metrics with fivefold cross validation in whole-lung radiomics analysis and multi-region radiomics analysis. Tables 7 and 8 show the results of the accuracy, sensitivity, specificity, and AUC of the prediction model with SMOTE in the whole-lung radiomics analysis and the multi-region radiomics analysis for the training and testing data. The differences in the accuracy between the prediction model with and without SMOTE were 1.7% and 0.03, respectively, in the whole-lung radiomics analysis and 2.1% and 0.03, respectively, in the multi-region radiomics analysis.

**Table 7:** Assessment of the predictive performance of the predictive model for training and testing data in the whole-lung radiomics analysis with the synthetic minority over-sampling technique.

	Training (%)	Test (%)
Sensitivity	65.8 (60.7–76.0)	59.7 (50.0–66.7)
Specificity	72.8 (60.6–80.8)	64.0 (57.1–71.4)
Accuracy	69.7 (66.7–78.4)	61.7 (54.2–66.7)
AUC	–	0.63 (0.59–0.70)

*AUC* area under the curve.

**Table 8:** Assessment of the predictive performance of the predictive model for training and testing data in multi-region radiomics analysis with the synthetic minority over-sampling technique.

	Training (%)	Test (%)
Sensitivity	86.5 (72.2–98.4)	84.8 (82.4–86.7)
Specificity	90.8 (84.1–97.7)	75.9 (74.0–80.0)
Accuracy	89.7 (82.7–98.1)	81.7 (79.2–83.3)
AUC	–	0.85 (0.80–0.90)

*AUC* area under the curve.

## Discussion

RP is a very important adverse event in radiotherapy for NSCLC, and if it can be predicted, it will be useful information for deciding the treatment policy, such as prescription dose and follow-up interval.

Previously, grade 3 RP was defined to serious adverse event. Dang et al. reported that grade 2 and grade 3 RP have different predictors^24^. Therefore, predictors of grade ≥ 2 RP require an original approach. Since immunotherapy has revolutionized the treatment of lung cancer^2^, it is very important to predict grade ≥ 2 RP because it prevents the continuation of immunotherapy. In the current study, we focused on finding a new method for predicting grade ≥ 2 RP. A previous report showed that background factors such as sex, smoking status, tumor location, age, and pulmonary comorbidity have been identified as potential risks^8,9^. Dosimetric factors, such as mean lung dose or V20, and several other DVH parameters have also been reported as the best correlated predictors of RP. Therefore, it is necessary to develop predictors that consider both types of factors.

Currently, radiomics approaches have been used to improve diagnostic quality or to predict treatment outcomes using medical images that have only been used for radiation diagnosis, treatment planning, and follow-up after treatment. Krafft et al. combined radiomics features extracted from whole-lung images with clinical and dosimetric features and significantly improved the RP model for grade 3 RP^13^. The cross-validated AUC for the model was 0.68. The current study compared the prediction model using the whole-lung radiomics analysis performed by Krafft et al. and multi-region radiomics analysis, which was proposed as a new method in the current study. We were able to reproduce the same degree of accuracy by using a method similar to that of Krafft et al., as though we predicted grade 2 RP rather than grade 3 for both the prediction model with and without SMOTE. Furthermore, we could improve the quality by using multi-region radiomics, which had superior accuracy, sensitivity, specificity, and AUC compared to whole-lung radiomics analysis for both prediction models with and without SMOTE.

In the multi-region radiomics analysis, the radiomics features of local intensity roughness and variation were selected from the CT images. A previous study showed that an increase in radiologic density within the irradiated lung was a predictor of RP. Thus, RP can occur due to local intensity roughness, and variation can increase the density on the CT image, which is an important factor in predicting RP. Moreover, the shape features were extracted from dosimetric-based segmentation, which reflects the dose-volume histogram metrics. Thus, dosimetric parameters are essential for predicting RP grade. For radiomics features in the dosimetric-based segmentation, the regions of the normal lung that received 60 Gy were selected as an important predictor in addition to the region of the normal lung that received 20 and 30 Gy. Although many reports emphasize the importance of the median^8^, the correlation between a high dose and RP has not been fully analyzed. We previously reported the importance of reducing high doses by analyzing NSCLC patients who received 3D-CRT or IMRT^10^. The current study supports the importance of a higher dose, as well as the median dose. We should reduce not only low and middle doses, but also higher doses to prevent RP. Although more features were selected in the multi-region radiomics analysis than in the whole-lung radiomics analysis, these features were based on evidence from a previous study. Thus, multi-region radiomics analysis can extract more effective predictors of grade ≥ 2 RP.

The problem with the previous radiomics analysis is that it is calculated from one value in the segmented region. RP mostly occurs in locally irradiated lungs. Multi-region analysis can extract radiomics features from the locally irradiated region, in addition to the whole lung. Hao et al. proposed a predictive model for distant failure using shell analysis. Shell analysis extracts features in the boundaries of the tumor, which allows us to detect its association with metastases within the microenvironment^14^. The current study also demonstrated that the radiomics features extracted in the multi-locally segmented region could be an important predictor of the classification of above and under grade 2 RP.

For feature selection with LASSO regression, all radiomics features extracted by texture analysis were used with a wavelet filter. Nie et al. reported that radiomics features with high-order filters and wavelets were significant predictors for differentiating focal nodular hyperplasia from hepatocellular carcinoma in the liver^25^. In the current study, all radiomics features with wavelet filters that were selected using LASSO regression were important predictors for the prediction of RP grade. The imaging filter can denoise, smoothen, or enhance edges and extract or eliminate a constant frequency. This leads to the elimination of redundant and effective factors for the prediction.

There are some limitations to the current study. Dosiomics and clinical factors such as smoking history, age, and chemotherapy could be integrated into the model to improve its prediction ability and robustness. Liang et al. proposed a prediction model for the RP grade using dosiomics analysis from the dose distribution for radiotherapy response prediction^26^. Although multi-region radiomics analysis has improved the prediction ability compared to dosiomics analysis, dosiomics analysis can extract spatial features such as local intensity variation of the dose distribution and the ratio of the low-dose region. Future studies will be performed using a combination of multi-region radiomics and dosiomics. Another limitation was that the current study used a dataset from a single institution of patients who underwent 3D-CRT. Feature selection was performed with LASSO regression via tenfold cross validation to prevent model simplification and overfitting and to select the optimal λ for the data. Moreover, we used SMOTE to balance the sample numbers after feature selection. SMOTE is a method of undersampling the majority class and oversampling the minority class^27,28^. The difference in the performance of the prediction model with and without SMOTE was significantly small, further enhancing the reliability of the results. In the future, we will conduct a larger multicenter study using both 3D-CRT and IMRT data to construct a highly versatile predictive model. Ambiguity in the definition of grade 2 RP may be another limitation, as in a previous report. In previous reports, the incidence of grade 2 RP varied considerably, and one of the causes may be that the definition differed between studies. In this study, the definition of grade 2 RP was based on CTCAE v.5.0. We tried to minimize subjective judgment by defining it as RP that requires treatment. Nevertheless, we successfully combined both individual patient background factors and dosimetric factors by analyzing RP with our new radiomics methods. Based on the prediction method developed in this study, it may be possible to reexamine the treatment plan by analyzing the images of the treatment plan and predicting the risk of grade 2 RP before the start of treatment.

## Conclusion

The developed multi-region radiomics analysis can help predict grade ≥ 2 RP for NSCLC after definitive radiotherapy. The radiomics features in the median- and high-dose regions and that of local intensity roughness and variation were important factors in predicting grade ≥ 2 RP.

## Supplementary Information

Supplementary Information 1.

Supplementary Information 2.
