# Supplementary material for: Prediction of radiation pneumonitis after definitive radiotherapy for locally advanced non-small cell lung cancer using multi-region radiomics analysis
Source: Sci Rep. 2021 Aug 10;11:16232. doi: 10.1038/s41598-021-95643-x (PMC8355298; doi:10.1038/s41598-021-95643-x)
Supplement: Supplementary file 1 — Supplementary Information 1. [file 41598_2021_95643_MOESM1_ESM.docx]

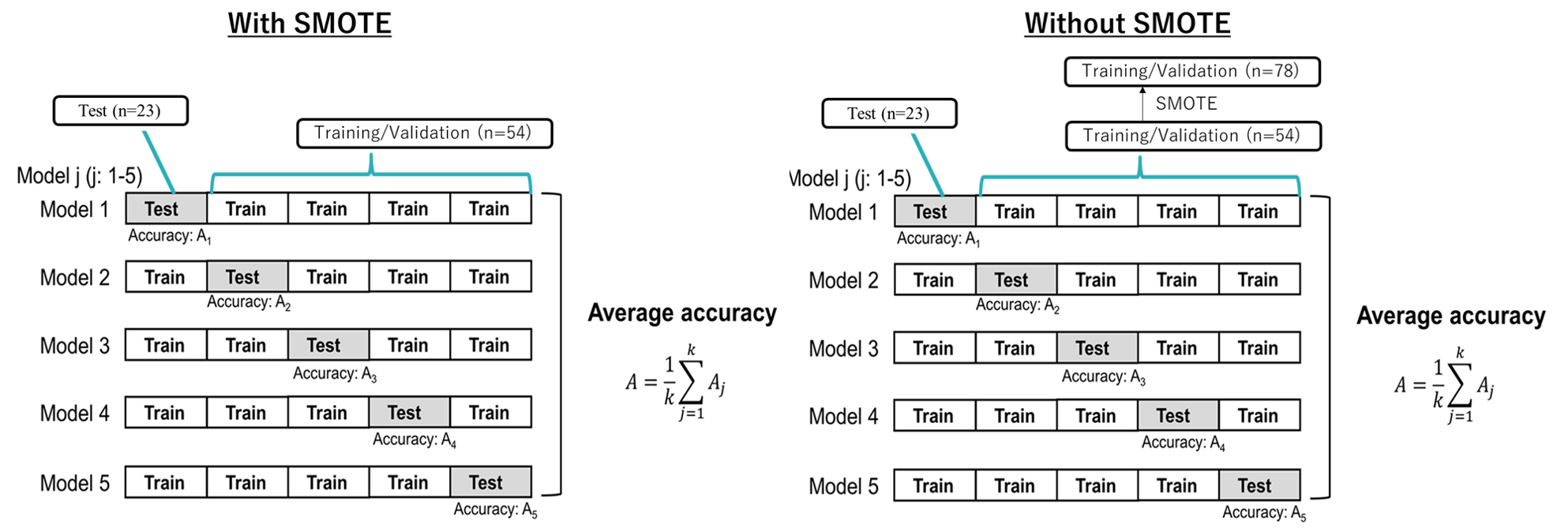


Fig. S1. Generation and testing of the prediction model without the synthetic minority over-sampling technique (SMOTE) (left) and with the SMOTE (right). The proposed neural network model with 5-fold cross-validation was built in the model training section.


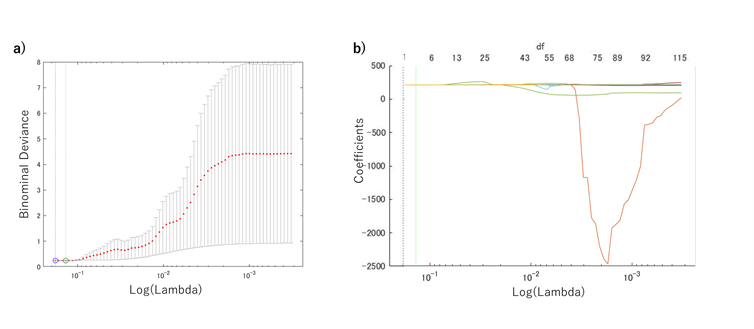


Fig. S2. Radiomics feature selection using the least absolute shrinkage and selection operator (LASSO) regression model in the whole-lung radiomics analysis. **a)** Tuning penalization parameter (λ) and minimum criterion in the LASSO model. The binomial deviance was plotted against log(λ). **b)** LASSO coefficient profiles of the 49383 radiomics features.


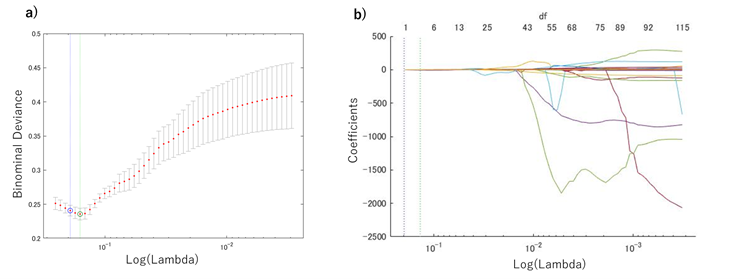


Fig. S3. Radiomics feature selection using the least absolute shrinkage and selection operator (LASSO) regression model in the multi-region radiomics analysis. **a)** Tuning penalization parameter (λ) and minimum criterion in the LASSO model. The binomial deviance was plotted against log(λ). **b)** LASSO coefficient profiles of the 49383 radiomics features.
