# Supplementary material for: Prediction of radiation pneumonitis after definitive radiotherapy for locally advanced non-small cell lung cancer using multi-region radiomics analysis
Source: Sci Rep. 2021 Aug 10;11:16232. doi: 10.1038/s41598-021-95643-x (PMC8355298; doi:10.1038/s41598-021-95643-x)
Supplement: Supplementary file 2 — Supplementary Information 2. [file 41598_2021_95643_MOESM2_ESM.docx]

Table S1. Feature type and associated features

| Feature type | Morphology -based | First order-based | Texture-based |  |  |  |  |
| --- | --- | --- | --- | --- | --- | --- | --- |
| Methods | Shape | Histogram | GLCM | GLSZM | GLRLM | NGTDM | GLDM |
| Feature name | Maximum 3D diameter | Interquartile range (IQR) | Joint average (JA) | Gray-level variance (GLV) | Short run low gray-level emphasis (SRLGLE) | Coarseness | Gray-level variance (GLV) |
|  | Maximum 2D diameter slice | Skewness | Sum average (SA) | Zone variance (ZV) | Gray-level variance (GLV) | Complexity | High gray-level emphasis (HGLE) |
|  | Sphericity | Uniformity | Joint entropy (JE) | Gray-level non-uniformity wholeized (GLNUN) | Low gray-level run emphasis (LGLRE) | Strength | Dependence entropy (DE) |
|  | Minor axis (MA) | Median | Cluster shade (CS) | Size zone non-uniformity wholeized | Gray-level non-uniformity wholeized (GLNUN) | Contrast | Dependence non-uniformity (DNU) |
|  | Elongation | Energy | Maximum probability (MP) | Size zone non-uniformity (SZ | Run variance (RV) | Busyness | Gray-level non-uniformity (GLNU) |
|  | Surface-volume ratio (SVR) | Robust mean absolute deviation | Idmn | Gray-level non-uniformity (GLNU) | Gray-level non-uniformity (GLNU) |  | Small dependence emphasis (SDE) |
|  | Volume | Mean absolute deviation | Joint energy (JE) | Large area emphasis (LAE) | Long run emphasis (LRE) |  | Small dependence high gray-level emphasis (SDHGLE) |
|  | Major axis (MA1) | Total energy (TE) | Contrast | Small area high gray-level emphasis (SAHGLE) | Short run high gray-level emphasis (SRHGLE) |  | Dependence non-uniformity wholeized (DNUN) |
|  | Surface area (SA) | Maximum | Difference entropy (DE) | Zone percentage (ZP) | Run length non-uniformity (RLNU) |  | Large dependence emphasis (LDE) |
|  | Flatness | Root mean squared (RMS) | Inverse variance (IV) | Large area low gray-level emphasis (LALGLE) | Short run emphasis (SRE) |  | Large dependence low gray-level emphasis (LDLGLE) |
|  | Least axis (LA) | 90^th^ percentile | Difference variance (DV) | Large area high gray-level emphasis (LAHGLE) | Long run high gray-level emphasis (LRHGLE) |  | Dependence variance (DV) |
|  | Maximum 2D diameter column | Minimum | Idn | High gray-level zone emphasis (HGLZE) | Run percentage (RP) |  | Large dependence high gray-level emphasis (LDHGLE) |
|  | Maximum 2D diameter row | Entropy | Idm | Small area emphasis (SAE) | Long run low gray-level emphasis (LRLGLE) |  | Small dependence low gray-level emphasis |
|  |  | Range | Correlation | Low gray-level zone emphasis (LGLZE) | Run entropy (RE) |  | Low gray-level emphasis (LGLE) |
|  |  | Variance | Autocorrelation | Zone entropy (ZE) | High gray-level run emphasis (HGLRE) |  |  |
|  |  | 10 percentile | Sum entropy (SE) | Small area low gray-level emphasis (SALGLE) | Run length non-uniformity wholeized (RLNUN) |  |  |
|  |  | Kurtosis | Sum squares (SS) |  |  |  |  |
|  |  | Mean | Cluster prominence (CP) |  |  |  |  |
|  |  |  | Imc2 |  |  |  |  |
|  |  |  | Imc1 |  |  |  |  |
|  |  |  | Difference average (DA) |  |  |  |  |
|  |  |  | Id |  |  |  |  |
|  |  |  | Cluster tendency (CT) |  |  |  |  |

Table S2. Features associated with imaging filters

| Feature type | LoG-based | Wavelet-based |
| --- | --- | --- |
| Methods | First-order statistic and texture of Laplacian of Gaussian (LoG). Filter width: fine, σ=0.5; medium, σ=1.5; coarse, σ=2.5 | First-order statistic and texture of wavelet decomposition. Decomposition levels: LLL, LLH, LHL, LHH, HLL, HLH, HHL, HHH. |
| Feature name | First-order features | First-order features |
|  | GLCM features | GLCM features |
|  | GLSZM features | GLSZM features |
|  | GLRLM features | GLRLM features |
|  | NGTDM features | NGTDM features |
|  | GLDM features | GLDM features |

Table S3. Patient characteristics

| Sex | Male/Female | 66/11 |
| --- | --- | --- |
| Age (year) | Median (range) | 68 (38–89) |
| PS | 0/1/2 | 61/14/2 |
| T factor (UICC-8^th^) | 1/2/3/4/x | 5/26/16/27/3 |
| N factor | 0/1/2/3 | 13/29/21/14 |
| Stage | IIA/IIB/IIIA/IIIB/IIIC/IVA | 0/13/23/32/7/2 |
| Total dose (Gy) | 60-65/66-69/70-74 | 14/12/51 |

PS, Performance Status.

Table S4. Patient characteristics for training/validation and test dataset

| Characteristic |  | Training/validation | Test |
| --- | --- | --- | --- |
| Sex (Range) | Male | 44–45 | 9–10 |
|  | Female | 18–23 | 1–4 |
| Age (year) | Median (range) | 69 (44–54) | 69 (38–54) |
| PS (range) | 0 | 40–45 | 17–22 |
|  | ≥ 1 | 17–21 | 3–6 |
| T factor (UICC-8^th^) | 1 | 3–4 | 2–3 |
|  | 2 | 19–20 | 8–10 |
|  | 3 | 15–17 | 3–5 |
|  | 4 | 26–27 | 9–10 |
|  | xx | 1–2 | 1–2 |
| N factor | 0 | 13–14 | 3–4 |
|  | 1 | 12–14 | 4–6 |
|  | 2 | 27–29 | 8–10 |
|  | 3 | 19–21 | 5–6 |
| Stage | IIA | 0 | 0 |
|  | IIB | 12–13 | 3–5 |
|  | IIIA | 23–24 | 8–9 |
|  | IIIB | 30–32 | 1–2 |
|  | IIIC | 6–8 | 1–2 |
|  | IVA | 0–2 | 1–2 |
| Total dose (Gy) | 60-65 | 11–13 | 5–7 |
|  | 66-69 | 12–13 | 14–16 |
|  | 70-74 | 50–51 | 3–5 |

PS, Performance Status.
